# Should they stay or should they go? A case study on international students in Germany

**DOI:** 10.1186/s40878-022-00313-0

**Published:** 2022-09-30

**Authors:** Sascha Krannich, Uwe Hunger

**Affiliations:** 1 Giessen University, Iheringstrasse 6, 35392 Giessen, Germany; 2 Fulda University of Applied Sciences, Leipziger Strasse 123, 36037 Fulda, Germany

**Keywords:** International student migration, Scholarship programs, Germany, Global south, Transnationalism, Development

## Abstract

International students are conceived as essential contributors to the development of their countries of origin after they finished their studies abroad. Political decision-makers of the countries of origin therefore take measures that students will eventually return to their home countries and bring back their gained knowledge and consequently contribute to development back home. However, is a return always the best way to contribute to development in the country of origin or can international graduates contribute equally from abroad or through their high mobility between different countries? This article aims to address this question on the basis of an intensive three years mixed-methods-based investigation in six countries – Germany as country of study and Colombia, Georgia, Ghana, Indonesia and Israel/Palestinian territories as countries of origin. We investigated a specific German scholarship program, which gives scholarships to international students from the Global South to study in Germany. Although a return to the country of origin is a precondition for the scholarship, our study indicates that not only *return migration*, but also *remains* and *circular migration* can create beneficial circumstances that former students practice diverse development-related functions and therefore contribute to the development in their country of origin in a specific way. Here, it is important to recognize that scholarship programs do not only offer the opportunity to fund studying abroad, but they can be also designed for the needs of scholars during, before and after their studies, which would also benefit their developmental contributions.

## Introduction

The impact of international student migration on their countries of origin is an under-researched subject in migration studies. International students are almost exclusively perceived from the perspective of the host countries in the Global North where they are seen as “ideal immigrants” (SVR, 2012) who are (mostly) relatively young and well-educated, and therefore, highly demanded skilled labor of the future (King & Raghuram, 2013; Pott et al., 2015).^1^ In contrast to skilled labor migrants educated in their home countries, international students are already well-integrated into the host society when they enter the labor market, because they have usually already learned the language, got used to everyday life, made friends, and eventually established their first professional networks during their studies. Therefore, many Western countries allow international students to stay after they finished their studies to search for a job. This should target particularly students of disciplines, which are in high demand on the labor market, such as in the STEM fields or medicine. Research studies about international student migration therefore focus often on the motives of students for studying abroad (King & Raghuram 2013, Thieme et al., 2014, Gëdeshi & King, 2019) and their choice for a specific country of study and field of study (Raghuram, 2013, Bijwaard & Wang, 2013).

However, it can be assumed that international students are also essential contributors to the development of their countries of origin after they finished their studies abroad. Political decision-makers of the countries of origin take measures that students will eventually return to their home countries and bring back their gained knowledge and create networks abroad to their country of origin one day, and consequently contribute to development in the country of origin. Therefore, many countries of origin of international students have even established their own scholarship programs for studying abroad obligating the students to return home after their studies.^2^ However, is a return always the best way to contribute to development in the country of origin or can international graduates contribute equally from abroad or through their high mobility between different countries? This article aims to address this question on the basis of an intensive three years investigation in six countries of a specific scholarship program and alumni network of a German stipend organization which gives scholarships to international students from the Global South to study in Germany. The goal of the program is that the students will gain knowledge and new experiences in Germany and then return home to introduce their new experiences in processes of development back home. The return to the country of origin is a precondition for the scholarship.

The underlying theoretical considerations of this program is related to the old brain drain theorem, meaning that the emigration of highly skilled migrants – especially medical doctors, engineers, scientists etc. – has negative consequences for developing countries (Logan, 1987; Mudende, 1989). From the perspective of dependency theories, brain drain was a strategy of industrialized countries to maintain dependency of developing countries (Bhagwati, 1976; Galeano, 1988). Emigration of the “best brains” led to the loss of important human capital with a negative impact on the socio-economic development of sending countries (Bhagwati & Rodriguez, 1975; Logan, 1987; Körner, 1999). However, this has changed slightly at the beginning of the twenty-first century. An abundance of research shows that emigration of highly skilled migrants does not have per se a negative impact on developing countries anymore, but that they can also take advantage of emigration. Once migrant elites have established themselves in the host country and got good positions on the labor market, they are able to transfer their gained experiences, acquired capital and know-how as well as networks back to the country of origin. Thus, they can be engaged in economic (Hunger, 2004, Portes & Martinez, 2019), political (Rother, 2017), social (Krannich, 2017) or cultural processes (Zhou & Lee, 2015) in their home countries. Sometimes they might do that even more effectively than they would have stayed at home and got no job. In this regard, researchers argue that an initial brain drain can transform into a brain gain (a benefit for the sending country) in the long-run (cf. Van Hear, 2003, Hunger, 2004, Thränhardt, 2005, De Haas, 2012, Faist et al., 2013, Portes & Fernández-Kelly, 2015).

Some theoretical papers and empirical studies already indicated – yet only based on smaller case studies – that international student migration can have these positive impacts on the home countries in the long run if students take over leading positions in the economy, politics, or academia in their home countries after their return from studying abroad (cf. King & Raghuram, 2013, Thieme et al., 2014, Gëdeshi & King, 2019). For instance, a survey of the German Academic Exchange Service (DAAD) revealed that over two-thirds of scholarship holders returned to their country of origin after graduation, and almost 90 percent of them perform a developmental relevant activity (DAAD, 2011). A study about students of a British scholarship and master program in Hong Kong, which includes a study period in Great Britain, indicated that knowledge and networks built by students during their studies were used later in their jobs (Leung & Waters, 2013). Similar trends evolved in the frame of a double master program between Dutch and Indonesian universities. Universities in China, Singapore, and Malaysia benefit also from returned students and scientists, who contribute essentially to academic capacity building by applying knowledge they gained abroad, particularly through the introduction of new research focuses and curricula (Ong & Chan, 2012). Other research studies arrive at similar conclusions about positive contributions of foreign alumni for the development in their countries of origin (see Schraven et al., 2011 about African graduates, Ghimire & Maharjan, 2014 about Nepalese students abroad, and Basford, 2014, Beech, 2014, Beine et al., 2014 about international students in general).

Another survey about Indian students and researchers (in Germany, France, the Netherlands, and Switzerland) showed that also these students contributed to the development in India by remigration (Tejada, 2013: 31). However, this study also showed that Indian students who went back after their studies had to face some barriers during reintegration into the labor market of their home country. Problems were the local working culture, bureaucracy, and bad public infrastructure (Tejada, 2013). And, there are still some studies that show the negative effects of emigration of highly skilled migrants, such as physicians from African and Asian countries, leaving to Europe or North America, cause enormous shortages of health care in home communities (Faist et al., 2013) showing, that it always depends on social and political determinants whether migrants can have a positive impact on their countries of origin (Massey & Durand, 2004).Against this background our study aims to contribute to the discussion on the “migration, transnationalism, and development- nexus” with the focus on student migration using a key example from Germany (see methods). Here we raise the question, if a return home after studying abroad is still the best option for regulating international student migration from the Global South, or are there other options how migrants can contribute to the development in their country of origin from abroad by now, particularly by financial remittances, know-how and capital transfers as well as social and political transnational networks. Since, there is no profound research on linkages between migration of students, their developmental impacts in countries of origin and the role of scholarship programs, we hope to provide also some insights on this aspect and share these considerations with the international community, given the fact that scholarships can be a good opportunity to steer international migration flows of students.

## Methods

As already mentioned, we analyzed these research questions using the example of a German scholarship organization and alumni program which was founded in the 1950’s and is one of the few organizations worldwide that aims to contribute to international development cooperation by supporting international students and researchers exclusively from the Global South. The scholarship program and alumni work are divided into five focus unit departments based on regions of origin: Africa, Asia, Middle East, Latin America as well as Eastern Europe/Caucasus. In total, over 10,000 international students and researchers from the Global South and Eastern Europe were funded to this day. Currently, the program supports about 450 scholars per year. Although this is a relatively small number compared to other scholarship programs, it is still remarkable due to its specific regulations. According to these policies, it is a requirement that scholarship recipients return to their country of origin right after finishing their study and scholarship program in Germany. This is a precondition of the scholarship contract, which participants must sign. Accordingly, the organization then covers the costs for the return trip as well as baggage and relocation costs. If recipients do not return to their country of origin after graduation, they have to pay back the full scholarship amount. With this restricted policy, the organization aims to prevent alumni from losing the connection to their countries of origin, and therefore, to foster a brain drain.^3^ However, as Germany’s immigration rules allow students for 18 months now to stay and look for a job in Germany, some of the students remain in Germany after graduation.

Germany as a country of study became very relevant for international student migration during the last decades. As mentioned above, Germany is the third most favored country of international students worldwide, only behind the United States and the United Kingdom, which makes it the most popular non-English speaking country (OECD, 2020). Numbers of international students in Germany increased over 50 percent in the last 12 years: From about 240,000 in 2008 to nearly 400,000 in 2019 (DAAD, 2020).

In our empirical analysis, we concentrated on the life courses of former students from five focus countries in the Global South: Indonesia, Israel/Palestinian territories, Colombia, Ghana, and Georgia. We chose these countries, because they are the main funding countries of the scholarship program located in different “development regions” of the Global South (Asia, Middle East, South America, sub-Saharan Africa, and Eastern Europe/Caucasus). In addition, the organization pursues different funding priorities in each country. In Indonesia, the organization traditionally supports students in the STEM subjects, which are – on the one hand – in strong demand in Germany and other Western countries. On the other hand, graduates of those subjects can also play a decisive role for the economic growth in developing and newly industrialized countries such as Indonesia. In Israel and the Palestinian territories, the organization has sponsored predominantly medical professionals and health scientists for a long time, as there was no opportunity to study medicine in the Palestinian territories until the beginning of the twenty-first century. Similar to the case of Indonesia, these are professions that are in strong demand all over the world and are equally critical for the development of the countries of origin. In Georgia and Colombia, there is a focus on legal studies, economics and social sciences among the scholarship holders. In Ghana, which strongly relies on its agricultural sector, students of agriculture are largely supported.

In the context of these five case study countries, we applied a methodical approach consisting of a mix of qualitative and quantitative methods in a *sequential mixed design* (Teddlie & Tashakkori, 2009). Here, we firstly conducted the qualitative data gathering and afterwards a survey, although the qualitative research was the main part of the study. In addition, we analysed official and internal documents and statistics about the scholarship program as well as conducted gatekeeper interviews with organization representatives and members of alumni clubs to gain essential information to conduct the fieldwork. We selected interviewees from three groups: alumni who returned to their country of origin (*returned students*), alumni who stayed in the country of study after graduation (Germany) (*remaining students*), and the ones who live and work in at least two countries (*mobile students*). Overall, we interviewed 135 alumni from these five countries for semi-structured in-depth interviews: 26 Palestinian alumni, 32 Indonesian alumni, 30 Ghanaians, 15 from Georgia, and 32 from Colombia. The numbers of interviewed alumni differ from country to country due to accessibility and responsiveness of conducted alumni as well as different quantities of funded alumni in the case countries. For instance, funded numbers of alumni are four times higher in Indonesia or Colombia than in Georgia. Furthermore, we selected primarily alumni for interviews of disciplines which were funding priorities of the scholarship organization in these countries, such as Indonesian alumni in the STEM subjects or Palestinian alumni of medicine. We did not differentiate particularly between male and female alumni. However, it was interesting to see that there was a relatively equal reply to our interview requests. About 53% (72) of the interviewees were males, and 47% (63) were females. Therefore, we will illustrate cases of male as well as female alumni in the results of this paper.

In addition, we conducted 50 complementary interviews and conversations with representatives of development-relevant institutions and organizations in countries of origin, including universities, businesses, courts, hospitals, German institutions like the *German Corporation for International Cooperation* (*GIZ*), the *German Academic Exchange Service* (*DAAD*), or the *Goethe Institute* (the central German cultural institution worldwide) as well as embassies, to investigate the networks of alumni as well as the student migration landscape in countries of origin (see Table 1). Finally, we did 18 complementary interviews with representatives of the scholarship organization to understand their program strategy and development aims. In total, we conducted 203 interviews.

**Table 1:** Conducted Interviews in Germany and the five case study countries

Country	Interview group	Numbers of interviews
Israel/Palestinian territories	Palestinian alumni	26
	Development-related organizations and institutions in Israel/Palestine	13
Indonesia	Indonesian alumni	32
	Development-related organizations and institutions in Indonesia	6
Ghana	Ghanaian alumni	30
	Development-related organizations and institutions in Ghana	10
Georgia	Georgian alumni	15
	Development-related organizations and institutions in Georgia	8
Colombia	Colombian alumni	32
	Development-related organizations and institutions in Colombia	13
	Representatives of the scholarship organization in Germany	18
	Total number	203

Source: table created by the authors

The interviews were conducted in English, German and Spanish, and took between 60 and 150 min. The interviews were mainly conducted in person during three to eight week-long research visits in the examined countries and in Germany between April 2016 and April 2018. Only a few interviews were done online (Skype), if we could not reach the interviewees physically. In all countries we used basically the same interview guidelines regarding former studies in Germany and current development-related engagement in countries of origin. Almost all of the interviews with alumni were recorded and transcribed, only a few were recorded in writing. Interview transcriptions were analysed by applying the *integrative analysis method* (Kruse, 2015). The observation protocols were analysed by applying *selective coding* (Strauss, 1991) to allocate important text passages to generated codes and central samples.

The quantitative part of the study is based on descriptive statistics and consisted of an online survey of all alumni and current scholars in the organizations’ database. With 569 responses from a total of around 3400 alumni, the response rate of was 17%. The survey participants were 57% males and 43% females. They were between 21 and 79 years old. The average age was 40 years. Most of them were Catholics (around 70%), others were Christian-Orthodox (14%), Protestants (4%) as well as Sunnis (2%) and Shiites (1%). About 4% did not belong to any religion. Most of them were from Colombia, Ghana or Indonesia. The survey was conducted in a time span of three months during winter 2017/18 in form of an online questionnaire sent by e-mail to all scholars and alumni. The comprehensive standardised questionnaire was chronologically divided into three main parts: (1) time before, (2) during the studies and scholarships in Germany (3) and time after graduation (in Germany, the country of origin or in another country). Questions focused primarily on the socio-economic situation before and while studying; funding, support and perception of scholarships in Germany; and professional careers, (social, political and economic) developmental engagement, alumni networks and other networks after graduation of alumni in the countries of origin as well as of studies.^4^ The descriptive analysis of the data provided a picture of frequencies and arithmetic means of socio-demographic characteristics (sex, age, marital status, presence of children, social background, study course, profession and income) as well as scholarships and developmental commitments of the alumni.

## Results

The field research revealed different forms of development contribution among former scholarship holders. According to the research design of our study and the above described different funding priorities in these countries, we focused on different areas of development relevant in these countries. In Indonesia, we focused particularly on the economic and technology system, because more than 60% of all Indonesian scholarship holders were funded in the STEM subjects. In the Palestinian territories (Westbank and Gaza Stripe), we focused on the health care system.^5^ In Georgia, we look predominantly on the development of the legal and political system, in Ghana, on the activities in the environmental and agricultural sector, in Colombia on the movement for peace and democracy. All of these different fields are main areas of development in these specific countries, which are supported by the states and international organizations. In regard to these various forms of development, we identified three different forms of migration patterns in each case study country: (1) *returned former students*, the ones that re-migrated to the country of origin after their studies, (2) *remaining former students* who have not returned (yet), and (3) *circular migrants* with permanent migration between different countries (the country of origin, Germany and/or other countries). Below, we describe selected findings of each migration form and development-related activities.

### Former students who returned to their country of origin

The majority of student migrants in our sample return upon graduation, which is not a surprise given the scholarship policies. In fact, about 80% of alumni return to their countries of origin. More importantly, more than three quarters of the respondents of our survey stated that they returned in order to contribute to their countries’ development. Almost half of the returnees have already supported an international development aid organization (either from Germany or from a different country) or have at least collaborated with one. In total, more than 90% of the alumni in the survey stated that they are satisfied with their decision to return to their country of origin. However, 60% of them mentioned that they returned because it was mandatory as part of the scholarship agreement. The return rates vary from country to country. In our case study countries, return rates of students after graduation differ significantly: Georgian and Ghanaian students show a high return rate of almost 90% each, mostly right after finishing their studies in Germany. Palestinian and Indonesian have an average return rate of about 65%, more than half of them not later than 12 months after graduation. In contrast, more than half of all Colombian students stayed in Germany (a relatively low return rate of below 50%), while over 80% of the ones who return to Colombia do it right after their graduation.

Our interviews revealed relevant examples in every case country where returnees contributed substantially to the country’s development. Almost all of the interviewed returnees worked in their study areas (about 95%). Most of them returned in the last 30 years, which had an impact on their career, because usually it takes some time to reach an influential job position. In Palestine, alumni found jobs mainly in private hospitals or settled down as self-employed doctors in rural areas of the West Bank. An example is a former scholarship holder, who returned about 20 years ago and is already for five years the head physician of the largest and best equipped pediatric hospital in Palestine, the Caritas Baby Hospital in Bethlehem.^6^ More than 50,000 patients are treated under her guidance every year. In her daily work, she is drawing not only on her knowledge gained in Germany, but also on a broad network of colleagues in Germany and the Palestinian territories. Frequently, she exchanges thoughts and findings about rare children’s diseases with physicians in Freiburg, Tübingen, and Hamburg. In doing so, for instance, radiographs are sent to Germany for consultation. Thus, “the one or other lives” could be saved, as the head physician explained. She pointed out: “That would not have been possible, if I didn’t study in Germany.” Here, she does not only refer to the mentioned contacts in Germany, but also to the gained expertise, which, as explained by the alumna, cannot be acquired in Palestine until today, but is much-needed in the West Bank. Only herself and some other colleagues, who studied abroad, could teach their knowledge to younger staff members in Palestine.

Other alumni, who studied medicine in Germany, became self-employed and opened their own private surgeries and clinics in Palestine after the return from Germany. This includes an alumnus, who did a three months professional training in orthopedy at a hospital in Kirchberg, Saxony, in 1999, and opened his own clinic Al Shifa in Beit Sahour, a neighboring town of Bethlehem, in 2002. In the meantime, almost 40 physicians and nursing staff are employed in his clinic, including specialists for radiology, orthopedy, and physiotherapy. Many of them did also study in Western European countries. Patients come from all parts of Palestine, because the clinic possesses the newest medical instruments and the high-quality treatment became well-known in the region. The founder funded the medical instruments by credits, which he took out during his more recent further medical trainings in Italy and the United States, which exemplifies the access to capital his transnational education grants him. The clinic focuses on the diagnosis of rare deceases, which requires a special know-how. The alumnus and owner of the Al Shifa Clinic said:

“We have specialized in the diagnosis of rare deceases, because, first of all, it is important to recognize and to understand the special form of decease before we can do the surgery. We can do that. Other doctors in Palestinian hospitals do not have the expertise, and they start to perform surgery even though they have misdiagnosed the wrong disease. In addition, there is no control of the work of doctors in state hospitals. People can die because of the wrong treatment, and there are no consequences for the doctors who were in charge of that. This is a big problem in Palestine” (interview with a Palestinian alumnus, Beit Sahour, West Bank, 2016).^7^

In addition, there are numerous other similar examples in Palestine, including two alumnae heading a private pediatric clinic, an alumna who opened her own dental clinic (both in Ramallah), or a radiologist working in a private hospital in East-Jerusalem.

The commitment of returned alumni in Colombia concentrates primarily on coping with various inner-Colombian conflicts as well as the support of the process of democracy in general, which raises hope of many alumni since the peace agreement in 2016. In this regard, many alumni consult Colombian political parties in the regeneration of the conflict and the peace dialog, or they advocate for the rights and interests of the victims of the conflict. For instance, an alumnus, who received a PhD in philosophy in Germany and returned to Colombia in 2005, consults Colombian politicians in the peace dialog with indigenous groups. In doing so, he draws upon a model of interreligious dialog between indigenous world-views and ideas on one side and Christianity in Latin America on the other side. The model is based on ideas of mutual respect, intercultural openness, solidarity, and communicative sensitivity. He developed this model in frame of his dissertation in Germany. In the meantime, the Colombian state took parts of this model and implemented it successfully in the dialog with indigenous communities in the north of Colombia, which suffered extremely under military conflicts during the civil war, but are widely self-governed now and started to cooperate with the Colombian government. Another Colombian alumna, who is a sociology professor in Bogotá, consults politicians and lobbyists in dealing and communicating with paramilitary organizations on the local level. She tries to implement a participative approach, which was not very common in Colombia to date, and which introduces more interests of paramilitary groups into the decision-making process.

Other Colombian alumni focus on the legal rehabilitation of the conflicts, like a lawyer who advocates for the rights of people with visual impairment. Many of them were victims of armed quarrels. Another lawyer – who got a PhD in Germany and returned in 2012 – works for the compensation and social reintegration of land mine victims. For that reason, she travels the whole country, particularly to rural regions, to educate not only local administrators, but also victims directly on-site about their rights. In the frame of an UN project, another alumna conducts workshops about trauma-focused coping for victims of the conflict in small villages in West Colombia. She helps especially women and children in dealing with their traumatic experiences, such as shootings or rapes. In addition, she also educates victims of rape about their rights and how to act legally against their perpetrators (interview with a Colombian alumna, Bogotá, 2017).

In Ghana, many returnees work for international NGO’s and companies in areas of environmental protection and agriculture to support Ghanaian farmers in introducing new and environmentally friendly forms of production. This is a main challenge in Ghana due to climate change and food supply issues (Asante & Amuakwa-Mensah, 2015; Nyamekye et al., 2018). In their work they use technologies that were developed by other alumni at German and Ghanaian universities. For instance, an alumnus at the University of Kumasi – who studied in the 1990s in Germany and returned to Ghana in 2002 – developed a new irrigation system for the cultivation of water intensive plants during dry season. This new irrigation system distinguishes itself from other conventional irrigation systems used in Ghana in that it is relatively easy to install and handle. Its operation is also very inexpensive, because it essentially consists only of an underground water pump and a water tank. Therefore, due to its affordability and easy installation, both small-scale farmers and bigger agricultural companies are implementing this irrigation system more and more.

Another Ghanaian alumna, who returned to Ghana in 2011, works for a German land survey company in Ghana. Currently, she manages a project about the creation of virtual maps for agricultural land. Directly after her master studies of geoinformatics and photogrammetry at the University of Stuttgart from 2008 to 2010, she did a half-year internship at this company. During that time, she called the companies` attention to market opportunities in Ghana. There were no companies like that one in Ghana at that time. The German company could not have gained ground in Ghana without the connections of the Ghanaian alumna to the Ghanaian Ministry of the Interior and to local administrative authorities. She made her point:

“The German company would not be in Ghana without my support and connections in Ghana. I told them about the opportunities here in Ghana, and I showed them the announcement of the project of the Ghanaian ministry. After they decided to apply for this state mission, I advised the company in the application process. I explained the important things they have to pay attention to in Ghana, about our customs, rules and bureaucracies, and what is important for the ministry, environmental conditions in Ghana, about challenges for doing land surveys in Ghana and with which partners we could work together on that project in Ghana. I already knew all these things pretty well from my colleagues here and because of my connections to Ghanaian authorities” (interview with a Ghanaian alumna, Accra, Ghana, 2018).

In Georgia, returned Georgian alumni contributed especially to the development of the legal system. This is the case, according to our interview partners, because jurists as experts with a well-grounded legal knowledge have relatively good job opportunities in Georgia. They are not only highly demanded by the Georgian state, but also by private companies and non-governmental organizations. Many Georgian law students were studying in Western Europe, especially in Germany (Tukhashvili, 2018). The reason is that the Georgian law is strongly based on Continental European law (Winter & Kalichava, 2019). After independence of the Soviet Union in 1991, when Georgia under its president Eduard Shevardnadze began to orient toward Western Europe, Georgian legislators attempted to create the new constitution on Western European law. Many former scholarship holders were also involved in several legal reforms in Georgia in the last years. An alumnus is dealing with issues of corruption in the frame of the Georgian criminal law. It is important to him to realize democratic legal principles and laws also in politics. Therefore, since 2011, in addition to his research projects, he works also in an assurance group for issues of corruption in the national parliament in Tbilisi. Here, he deals with cases of corruption of members of the national parliament across all parties and with the legal clarification of these cases. He benefits from his legal knowledge he gained in Germany and its practical implementation in his work. The Georgian criminal law and the laws for fighting corruption, according to the alumnus, were also largely copied from German law.^8^ His good German language skills help him to work with German criminal law books to find explanations in uncertain cases. In doing so, he gets new ideas how to formulate specific parts in the Georgian law in clearer way. Teaching at the Caucasus University in Tbilisi, he realized that there are almost no deep and comparative textbook in the Georgian language. Therefore, he decided to write a textbook on criminal law, which describes existing law on the basis of concrete case examples. His dissertation, in which he analyzes multiple cases of German as well as Georgian criminal law, would provide a lot of material for his textbook.

Similar cases of returnees could be also found in Indonesia. Many of them obtained leading positions in the Indonesian economy or started their own businesses. For instance, an alumnus who studied architecture in a master program at the institute of technology in Karlsruhe in South Germany, became self-employed as an architect in Jakarta after his return in 2009. Currently, he takes part in the construction of the biggest church in the north of Jakarta with over 2000 seats. Due to the fact that his small business with only two employees does not have enough capacities, he collaborates closely with three other architecture companies to realize this church project. He designed the ground plan for the church, and supervises the construction work for it. The project should be finished in 2022. Already during his studies in Germany, he developed a sense for entrepreneurship in exchange with other German and Indonesian students, and built up a network with other Indonesian entrepreneurs.

Another alumnus became also a businessman in Jakarta. However, after his engineer studies at the RWTH Aachen in 1996, he worked at first for six years at different companies in Indonesia, including larger international corporations from the United States, to gain work experience as an employee. Nonetheless, already during his studies, he had the plan to open his own business. In 2002, he founded a telecommunication company, which sells pre-paid phone units in over 40,000 businesses in Indonesia. He has over 20 employees by now. In the future, he wants to expand his company and enter the cellphone market.

Among alumni who returned to their home country also exists a high level of mobility: almost 70% of them came back to Germany at least one more time after they returned home, 34% even came back every one or two years after their return, and 14% even two or more times every year. Nearly half of them usually stay only for a month or shorter during their visits to Germany, and 15% stay between one and six months, mostly for research (40%), but also for business (5%) or to work with state institutions (5%). These frequent short-time visits stand for a distinct type of mobility (see more details below).

### Former students who remained in Germany after studies

Several alumni also remained in Germany after finishing their studies. However, this doesn’t mean that these former students are “lost” for their country of origin as assumed in earlier studies. In contrary, we found that also this group contributed intensively to the home country’s development through transnational networks as well as know-how and capital transfers from Germany to their countries of origin in a variety of ways. For instance, a Palestinian alumnus – who studied human medicine with a German scholarship at the university in Regensburg until 2011, and works since that time as a medical specialist at a hospital in a bigger German city in the Ruhr area – established an exchange program with a clinic in Palestine. In the frame of the program, he invites Palestinian doctors for a six months medical training in Germany every year. The invited doctors can be trained in all medical fields, particularly at medical tools and instruments, which do not (yet) exist or were only recently introduced in Palestinian hospitals, but where not enough know-how and experience exist to apply these tools. Here, several German doctors are involved, who pass on their special knowledge to the guest doctors. In doing so, Palestinian physicians gain valuable know-how for their work in Palestinian hospitals. The exchange program is free for Palestinian doctors. By transferring the knowledge permanently back to Palestine, the achieved impact on the development of Palestinian hospitals is much bigger than his (one-time) return:

“These medical trainings have a multiplier effect. The doctors acquire a valuable expertise, which they could not get during their education in Palestine because of bad facilities and equipment. After their return to Palestine, they give this expertise to their team of four, five co-workers. Thus, I support over 20 doctors and nursing staff. If I would return, I would only support my team, if I would actually find a position as a doctor” (interview with a Palestinian alumnus in Germany, Skype, 2016).

Another former scholarship holder became a family doctor in the German state of Saarland after he studied medicine at the University of Saarbrucken from 1999 to 2006. He founded his own medical practice in Homburg in 2013. In addition, he constructed a transnational network of support between the Gaza Strip and Germany together with his brother and other doctors in Germany. In a sponsorship program, this network collects money from patients in Germany for children in need in the Gaza Strip.^9^ These donations cover costs for treatment of these sick children. So far, over 50 sponsorships were realized. In some cases, doctor’s bills over 200 Euro were paid by patients in Germany for the medical treatment of children in Palestine. Another Palestinian physician organizes relief projects from Germany to support Palestinian doctors in Gaza:

“I can help them more, because I live in Germany. Here, I have a higher income, more security, and a faster access to working partners. From Gaza I could not take care of the aid projects for doctors there. It would not help anyone, if I would go back to Gaza. Here in Germany I also have a faster Internet, telephone, and a good infrastructure. That is all not the case in Gaza” (interview with a Palestinian alumnus in Germany, 2016).

Another alumnus from Palestine – who is currently a professor for clinical psychology with a focus on therapy research at the University of Applied Science in Göttingen and researches in the area of fear therapy and treatment of traumata of refugees – is trying to build a research network with several universities in the Middle East. In particular, he aims to develop a very new branch of psychological therapy research and consolidate its support during research stays in the region, but this remains difficult under current political and social conditions.

In Colombia, many alumni who did not return after graduation from Germany are nevertheless engaged in the peace and pro-democracy movement. A Colombian alumnus organizes frequently trips to Colombia with a group of German and Colombian students and university professors, who are appointed there as mediators in the conflict between locals and guerilla groups. Here, he works closely with other alumni in Colombia. Another alumna is active in the German-Colombian peace and conflict research network, called *Instituto Colombo-Alemán para la Paz* (CAPAZ). She is advocating for the concrete implementation of the Colombian peace treaty in a small village in Northern Colombia. In detail, it is about the question how the municipal voting right is realized on-site and to what extent the local population perceives their political representation on the local level. In this regard, an intensive exchange evolved between members of the research network in Germany and Colombia. Despite manifold commitment and sporadic progress, most alumni are rather skeptical about the success of the peace process and conflict rehabilitation.

Ghanaian alumni – who stayed in Germany after their studies – are also professionally engaged in a sustainable ecologically development in Ghana. For instance, an alumnus is working in the frame of a three years project at an international NGO called *Local Governments for Sustainability* in Bonn. The NGO advocates for sustainable policies in communities worldwide, including Ghana. Particularly for Accra, he develops a strategy to reduce emission rates (especially caused by cars). The strategy consists mainly of a combination of more public transportation and a traffic-reducing infrastructure. To realize this project, he cooperates with responsible communal agencies in Accra as well as several federal ministries and organizations, including the health ministry and the Ghana Health Service. He collected the first concepts and ideas for this project already during his studies. In his master’s thesis in the degree program *Geography of Environmental Risks and Human Security* at the University of Bonn, he focused on the impact of climate change on the cocoa production in Ghana, and developed an ecological approach for crop increase in cocoa cultivation farming. His work shows that many farmers did not find adequate instruments to face changing cocoa growth due to rising temperatures and low rainfalls.

Another alumnus became self-employed in Germany: Beside his studies in Leipzig, East Germany, he opened an online shoe store in 2016. In this online store, he sells shoes made of second hand and recycled clothes from Germany. He buys the clothes in second hand stores in Accra and sends them to an employee in Ghana, who takes care of the shoe production with six other employees (all are Ghanaians). The shoes are made in a modern design for a young target group (males as well as females). That little company founded and headed by an alumnus is a good example for a small transnational business, which creates also jobs for young people in Ghana (currently seven between 20 and 24 years old).

In Georgia, some of the recent reforms in their legal system can also be traced back to former scholarship holders who stayed in Germany. Several areas within the Georgian constitutional and criminal law have been reformed with the contribution of former students from Germany. For instance, one alumnus consults the Georgian Ministry of Justice in Tbilisi. Another one works for the legal committee of the Georgian parliament. Because he stays in Germany, he has fast access to German law literature as well as professors and judges:

“It is really like my colleagues from the Ministry of Justice in Georgia contact me regularly and ask about how specific laws and rules are look like in Germany. Usually, I go then to the library and look to the right textbook or law book, and then I translate paragraph by paragraph. Then I send my translation to colleagues in Georgia. If I would be in Georgia, I would not have access to these resources” (interview with a Georgian alumnus in Germany, 2016).

A current Indonesian scholarship holder is working in the frame of his PhD at TU Dresden on the development of a new building technique for multi-story buildings, which would not only be made of steel and concrete, like is currently common in Indonesia, but also of wood and synthetic materials. This new building technique aims to make high-rises safer and preserve them from quick collapses in earthquake prone regions of Indonesia. He works not only with colleagues of TU Dresden, but also with the Bandung Institute of Technology, who want to realize this new technique in cooperation with an Indonesian company in Indonesia (interview with an Indonesian alumnus in Germany, 2017).

In every case study country, former alumni living in Germany also send money back home to their family and friends, or even business partners. Our survey revealed that currently about 30% of the survey respondents have sent money to their country of origin. About half of them state that they are sending money regularly. The average amount of money sent back home is between 100 and 200 Euros per month. In addition, according to the online survey, more than 40% of alumni who stayed in Germany after graduation traveled back to their home country for occupational reasons. Basically, over 40% of them are in permanent or casual professional contact with colleagues in country of origin.

### Circular migrants

Besides the students who returned or remained, there was also a smaller group of students who stayed mobile after their graduation in Germany, which means that they moved back and forth between Germany and the country of origin or moved on to another country or circulated between various countries (circular migration). The scholarship program of the organization supports long-term mobility in itself, particularly in academia and sciences, in that it is possible for students and researchers to apply more than once for a scholarship during their academic career – for example in the frame of a master’s program, PhD program, postdoc, or other short-term research stay. However, this mobile group is much smaller than the other two student groups. According to the online survey, 12% of the respondents received a scholarship twice to study or research in Germany, 4% even three times and 3% four times or more. For instance, a theology professor from Georgia has been going to Germany every two or three years for a short research stay of about 2–6 months to work at the university or in the archives on medieval history of religion. He has been doing that already for 16 years. This academic long-term mobility would not be possible without the specific support of the organization. Through these multiple stay scholarship programs, the organization aims to support the academic development in these countries through long-term mobility and hopes that funded researchers are able to build networks and joint research projects.

Other alumni also build networks between their countries of origin and Germany, which in some cases also extend far beyond Germany and their country of origin. An Indonesian female lawyer, for example, who gained a PhD on a scholarship in Germany and returned to Indonesia in 2010, is now vice dean of the law faculty at Atma Jaya University in Indonesia. She established a dense research network between Europe and Asia. In this framework, she is permanently in Europe, most recently for a two years postdoc stay in Belgium, where she worked with competition law in Europe.

We could also find circular mobility with regards to business activities among the researched group. Here, the long-term mobility among Indonesian alumni, as mentioned above, stood out due to their relative economic success. A prominent example is the founder of a technology company, which produces tools and home appliances. He studied architecture in Mainz and Braunschweig in the 1980s, and after his return he became self-employed and brought know-how on environmentally-friendly production methods for tools and important business contacts with German companies back to Indonesia. Over the last decades, his enterprise has become a leading company in its branch and one of the ten biggest in Indonesia. He prefers to employ other alumni in his company, because of his good relationship to the scholarship organization and conviction in their education and reliability. Furthermore, their working and language skills allow them to work for a specific amount of time in Germany and other countries abroad again to establish new business networks and markets for the Indonesian company. In this way, there emerged a kind of long-term mobility chain of alumni of the scholarship organization and his company. Part of that includes a younger alumnus who developed a new impact drilling machine for this company, which he learned during his studies of automation engineering at the RWTH Aachen. The company uses the foreign contacts of its employees to build a dense global network between German universities, the company, and beyond, particularly to connect STEM graduates between both countries. An alumna who is working at the company explained:

“Already before I started my studies in Aachen, I knew about former students who work for this company. They explained to me how I could also become employed that and that it is important to gain professional skills and learn about German culture in Germany, because we need these skills in our job, because there will be a lot of travel between our countries and beyond. […] After my return to Jakarta, I got a job at this company and I have to go to Germany several times a year to talk to business partners. […] That is quite stressful, but very important for my career and for the company” (interview with an Indonesian alumna in Jakarta, Indonesia, 2017).

Another alumna from Indonesia works as a mechanical engineer at a consulting firm in Munich. She already worked part time during her studies at this company. Back then, she recognized that the company could employ the former student for potential projects in Indonesia in the future. Directly after her graduation, she was asked by the company whether she wants to work for the company in full time and was hired immediately. She works permanently in Indonesia for several weeks or months. For a project on the Indonesian island Sumatra, she was a whole year on-site to consult an Indonesian company about the construction of an evacuation building in a tsunami area on Sumatra. In doing so, she focused on security policy, the construction of emergency exists (which should be handicapped accessible and barrier-free), signposting, and finger boards. Here, she learned a lot from the instructions in Germany, which are very precise and well developed. In Indonesia, such safety regulations do not yet exist for the most part. The project was financially supported by the Indonesian government as well as various organizations in Germany and Austria.

In the other case study countries, we could find similar examples of circular migration. For instance, Georgian alumni who work for the Georgian state or German organizations in Georgia travel frequently to Germany in the frame of their jobs. A Georgian alumna – who returned after her law studies in Germany to Georgia in 2014 – works for German development organization in Tbilisi. In the frame of development projects in Georgia, she also works several months a year in Frankfurt/Main or Berlin to coordinate projects of NGOs who support democratic developments like free speech or civic engagement in Georgia. In doing so, she does not only help to transfer democratic ideas from Germany to Georgia, but also helps the German organization to understand the specific political circumstances in Georgia. “I always explain to my German colleagues that Georgia is still on the way to establish a complete democracy, and that Georgians still argue if the German or the American form of democracy fits better to Georgia” (interview with a Georgian alumna in Tbilisi, Georgia, 2016).

## Discussion and conclusion

In a nutshell, our study indicates that all three forms of initial student migration – *return migration, remains and circular migration* – can create beneficial circumstances that former students practice diverse development-related functions and therefore contribute to the development in their country of origin in a specific way. Know-how and idea transfer are certainly a main function here, as described above. As researchers, they operate as multipliers by introducing new research and learning methods, and pass them on to their students. As doctors, they invite colleagues from their countries of origin to Germany for further training, or as entrepreneurs, they create new jobs, etc. In reverse, alumni also contribute to advance the German economy as well as German sciences and academia, particularly through their activities as head physicians, professors, or businessmen. In many cases, alumni are in leading positions and/or fulfill pioneer work. This relates to male as well as female alumni. For instance, they are involved in the foundation of new universities, or introduce new diagnostic procedures and therapy methods as physicians. Here, alumni contribute to important structure formations and social differentiation processes in relevant social segments like health, legislation, politics, economics, or sciences (see Bommes, 2003 on migration and social differentiation).

Equally important is their function to build bridges and establish networks. In this context, former students have also an anchor function. That means students do not only build bridges between Germany and their country of origin and link both countries through their travels and activities, but they can be also requested systematically as contact persons by other organizations to gain ground in these countries. These organizations include – as described above – companies, NGO’s as well as state institutions like universities or hospitals (in the frame of research projects) and international development organizations. This plays also a central role in the academic field, because knowledge can not only be acquired and transferred, but also produced in networks (Phelps, 2012). Although age matters in these processes and it takes some time before alumni stand on their own feed, it is remarkable that many alumni are still relatively young, such as a 31-year-old judge at the Supreme Court in Colombia, a 28-year-old electrical engineer who is in charge for the development of boring machines in one of the largest tool companies in Indonesia, or a young alumna at the University of Ghana in Accra who is a leading researcher in the development of sustainable agriculture methods in Ghana. This shows the enormous potential of student migrants, because many of them are still at the beginning of their professional lives and can certainly achieve even higher positions in the future.

The study made also clear that development promotion can be realized through various forms of migration, such as return migration as well as remains (through transnational social networks from abroad) and also through circular migration. Under the circumstances of a complex and highly connected world today, there is no clear answer to the question, which form of migrant regulation is the best under development-related viewpoints. Student migration in fact plays in all forms an important role for international development cooperation between Germany and the countries of origin, which goes beyond the old *anti-brain drain theorem* (prevention of migration) and involves a long-term understanding of development from brain drain to brain gain and brain circulation. However, it can be criticized that the current discourse on international students is almost exclusively about the gain of skilled labors in the countries of study in the Global North. The role of international students as *motors of development* and *change agents* in the Global South is almost nonexistent in the scientific discourse^10^ and the political arena. It can be stated that international mobility of students and alumni has a big potential, yet could be much better coordinated by politics allowing international students more degrees of freedom than a strict prohibition of further migration and an obligation of return after graduation.

As our study illustrated, especially scholarship programs do not only offer the opportunity to fund studying abroad, but can be also designed for the needs of scholars – during, before and after their studies:

Firstly, the ones who want to re-migrate to their country of origin (*potential returnees*) have to get better support for their return, including job mediation and specific re-integration programs. It needs more re-integration support after the return to their country of origin, because re-integration bears the most challenges for students (search for job and accommodation, infrastructure, culture, etc.) (cf. Guissé & Bolzman, 2015, Lin & Kingminghae, 2017), as we also learned from our study. In order to create attractive job perspectives after studying, either in academia or in private enterprise, which are the most important drivers for decision making of international students (Raghuram, 2013, Gëdeshi & King, 2019), the scholarship programs should be accompanied by financial support for the country of origin. In this sense, a university place abroad could be, for instance, co-funded by a company subsidiary in the country of origin with the perspective or guarantee of a job at this company directly after graduation. That could be also included in the frame of bi- or multinational companies, which enable to work in the country of origin as well as in the country of study or another country after studying abroad, and which is based on a (possible) job guarantee afterwards. Furthermore, this approach can also include the interests of the countries of origin, for instance, by systematically promoting the fields of study and research projects, which relate to developmental core areas in the country of origin, such as agriculture and environment in Ghana, the peace and democracy process in Colombia, or the development of the technology sector in Indonesia. That can be much better coordinated with the support of local and federal organizations on-site, including state development agencies or state ministries.^11^

Secondly, the ones who want to stay in the country of study (*potential remains*) have to get also a better assistance for integration into the labor market (as well as other integration-related measures) and support of their transnational activities and networks in their countries of origin. Until today, this group is almost completely ignored by the scholarship organization as well as by other development organizations in Germany. However, as aforementioned, these migrants also do an important job in terms of development cooperation. For instance, the creation of an alumni association in Germany in the frame of scholarship programs – like alumni associations in countries of origin, which already exist in our case study countries – could support networking among migrants who stayed in Germany as well as in other countries. Here, scholarship organizations could also work closely with organizations founded by migrants from these countries. Some alumni are already active in migrant organizations, which are involved in developmental processes in the country of origin. For instance, Colombian alumni who are active in political migrant organizations, which advocate for the peace process in Colombia, or Indonesians who are members of entrepreneurial organizations to enhance transnational business relations with Indonesian companies.

Finally, it is necessary to create better conditions for those who want to practice circular long-term mobility (*potential circular migrants*) in the frame of alumni programs in cooperation with universities, businesses, civic organizations and/or states. This could be achieved by the introduction of long-term research and business visa – which would be accepted by all involved countries – multiple entry visas in the country of study (and other relevant countries if possible), low travel barriers, low bureaucratic immigration costs, and international acceptance of academic degrees as well as fast knowledge and language tests to become accepted to study or work abroad, as already demanded by the *Global Compact for Migration* of the United Nations in 2018. However, there is no coherent idea yet on how to support and manage international student migration on the global level (UN General Assembly, 2019). Especially student migration in the frame of scholarship programs is a good form of migration, which can be well-organized and regulated in the frame of international structures, a so-called *global migration governance* (Betts, 2010). Here, scholarship programs for international students could be coordinated either in a binational or multinational frame – supported by the country of origin as well as the country of study under support of specialized international institutions – or solely in the context of international institutions, such as the *International Organization for Migration* (IOM). In this sense, international student migration can be governed as an impetus of a *long-run migration cycle*, starting with studying abroad and followed by either working abroad or in the country of origin (after re-migration), which could lead to circular migration in the frame of a job (labor migration) and transnational networks. At the beginning of this cycle, specific fields of study, in which skilled workers are needed in the country of origin, can be funded in the frame of scholarship programs. For instance, in the STEM fields in Indonesia or in agricultural areas in Ghana. Here, scholarship programs can be always adapted to the specific needs in certain countries of origin.^12^

After all, it needs to make sure that the sponsorship of international student migration does not lead to a one-sided flow into the countries of the Global North, and therefore, to a new postcolonial dependency of the countries of the South from the academic knowledge of the North. Above South-North dimension, international scholarship programs should rather also largely fund international studies in countries of the Global South and support long-run circular exchange of know-how between the North and South. Because the Global North can also learn a lot from the South without creating new postcolonial dependencies (Kerner, 2017 based on earlier considerations of Fanon, 1963 and Said, 1978). A Ghanaian alumnus from our study put it in these words: “There are many things that Germany can learn from Ghana. The most important thing is to see the interconnections between life, work, family, and the environment. It is important not only to value work, but also to value family” (interview a Ghanaian alumnus in Kumasi, Ghana, 2018).

These considerations may contribute to the broader discussion about international migration and development. Our case study illustrates on the one hand the general challenge to bring together the above-mentioned interests of the countries of residence (mainly to gain highly skilled labor force) and of the countries of origin (primarily to win leaders for development). And on the other hand, it also shows the great potentials of a fruitful cooperation between immigration and emigration states. That goes far beyond the question of student migration, it includes all kinds of migration from labor, family, to refugee migration. Liberal, open societies from the Global North can help to create favorable social and political circumstances to make free flows of circular migration possible, and can help to foster the potential developmental contributions by supporting former international students who stayed in their country in their developmental activities. In the light of other global challenges like climate change and pandemics cooperation of states will be a fundamental precondition to maintain a free movement of people. The current COVID-19 pandemic led to particular forms of immobility among international students and put many of them into precarious situations (Raghuram & Sondhi, 2021). Scholarships for students could help here as well, if they provide support and extra funding in times of hardship.

## Data Availability

The dataset used for this manuscript is deposited and available for the public if needed.

## Footnotes

1 Although international students are still a relatively small migrant group of ca. 8 million compared to refugee migrants of 78.5 million (UNHCR, 2020) and labor migration of more than 150 million people (ILO, 2019), numbers of international students increase fast in all countries where they are recorded. Only in OECD countries, numbers of international students almost doubled in the last 10 years, most of them studying in the United States (currently more than 20% of all international students), followed by the United Kingdom (11%), Germany (7%), France (7%) and Australia (6%) (OECD, 2020)). The main countries of origin are China and India (UNESCO, 2021). However, there are also many students from other Southern countries including Africa, Asia and Latin America (UNESCO, 2021). Although the numbers are difficult to determine, also South-South student migration is rising almost as fast as South-North migration, in some regions even faster (like in sub-Saharan Africa or China as a main destination country for students in the South (Sun, 2020).

2 This includes countries of origin of the largest diasporas worldwide, such as China, India and Mexico. Thus, they want to take advantage of good education opportunities in Western countries for their future leaders and their impact on the development of the country after their return. For instance, the government of Georgia established scholarship programs in close cooperation with domestic organizations and state institutions which offer jobs to Georgian students and alumni abroad to attract their return (Krannich & Hunger, 2022).

3 However, there are exceptions to this rule, if students would like to stay for another couple of months or even one or two years after graduation in Germany for the purpose of an internship or a postdoc project. Here, the organization is willing to support long-term mobility of scholarship holders and alumni as long as it does not lead to permanent absence of the alumni from their home country.

4 Before we launched the online survey, we checked the questionnaire in a first pretest with five test persons. After the evaluation of the first pretest and a profound revision of issues and questions, we did a second pretest with five test persons to test the final eligibility and exactness of the online survey for the study.

5 This includes medicine, dentistry, pharmacy, psychology, health sciences, public and global health.

6 The alumna wrote her PhD thesis about *New therapeutic approaches in the treatment of newborn babies* at the University of Munich in the 1990s. Afterwards, she was trained as a medical specialist at the University of Würzburg with the support of a German scholarship, and later, after a short term employment in Bethlehem, in the field of intensive care at the University of Freiburg with a second scholarship in 2003.

7 All quotes from transcribed interviews were translated and paraphrased by the authors for publication.

8 See Winter and Kalichava 2019 about the development and transformation of law in Georgia.

9 Alumni are also engaged in the development of the Gaza Strip. Due to the fact that many alumni did not return after studies because of the extremely unsecure situation in Gaza, they try to improve the situation in Gaza from Germany over contacts and transnational networks.

10 The scientific discussion is mainly about remittances (de Haas, 2012), the support of transnational networks of migrant organizations (Portes & Fernández-Kelly, 2015), and since recently the assistance of re-migration of refugees (Loescher et al., 2016), but not about international students.

11 In some cases, this has already started, but only through the self-initiative of migrants. Some former students founded alumni clubs (with support of the scholarship organization) in countries of origin to facilitate social and professional activities of former scholarship holders and to help returnees to reintegrate into the society and labor market of the home country. For instance, a regional alumni club in West Africa cooperates closely with the German GIZ/CIM (Center for International Migration and Development) to include newly returned alumni into the CIM re-integration programs, including funding and job search. In Indonesia, the alumni club organizes learning opportunities, job fairs, and helps returnees to access job positions in academia. In some cases, these events have even helped alumni to start their own businesses in close connection with German universities and companies.

12 Smaller structural elements of a global student migration governance on the UN level do already exist, including the UN university network (UNU) with its own scholarship program. However, these institutions lack sufficient funding and purposeful cooperation with affected states, universities, and other relevant actors (United Nations, 2020).
